# Using Community Pharmacy Immunization Screening Forms to Identify Potential Immunization Opportunities

**DOI:** 10.3390/pharmacy7040160

**Published:** 2019-11-26

**Authors:** Albert T. Bach, Jeffery A. Goad

**Affiliations:** School of Pharmacy, Chapman University, Irvine, CA 92618, USA; abach@chapman.edu

**Keywords:** vaccines, pharmacy, screening, missed opportunities, indications

## Abstract

Immunization screening forms are completed for each patient that is to be vaccinated in the pharmacy. Screening forms contain demographic and health questions, which are used to determine if a patient is contraindicated to receive a vaccine. The objective is to determine if patient responses to questions on these forms can be used to identify potential vaccine indications. De-identified data was retrospectively collected from 11 community pharmacies in California and Michigan that included basic demographics, answers to immunization screening questions, and vaccine(s) administered during that visit. The Advisory Committee on Immunization Practices (ACIP) recommendations were used to forecast vaccine needs using the limited demographic and health history available from the screening forms. Descriptive statistics are presented, characterizing patient demographics and health condition-based recommendations, and the percentage of patients in a pharmacy population that may have potential indications for additional vaccines. Data were collected from 8669 pharmacy vaccine screening forms. Using the patient’s date of birth on the screening form, 10% (*n* = 759) and 34.6% (*n* = 2615) of patients receiving vaccines at the pharmacy may be indicated for the zoster, or both the zoster and pneumococcal vaccines, respectively. Screening form questions that inquire about medical history are also able to identify 13.9% (*n* = 977) of patients with a potential need for pneumococcal vaccines. Our data indicate that pharmacists can identify potential immunization opportunities proactively by using their immunization screening form, not only to identify contraindications, but also indications.

## 1. Introduction

One of the barriers to increasing adult vaccination rates and reasons for missed opportunities for vaccination is that many healthcare professionals do not routinely assess their patient’s vaccination status [1]. Pharmacists across the United States (U.S.) have been vaccinating since the 1990s. However, pharmacists have traditionally been reactive vaccinators, waiting for patients to ask for a vaccine instead of proactively assessing them for vaccine needs. Pharmacists may also only promote a subset of vaccines that are commonly reimbursed for administration in the pharmacy by third party payers or prescription drug plans (e.g., zoster and pneumococcal vaccines) [2]. The National Vaccine Advisory Committee and the Centers for Disease Control and Prevention (CDC) advocate that all healthcare professionals be proactive in assessing immunization and providing a “strong recommendation” to their patients for needed vaccinations, as this is the strongest predictor of patients getting vaccinated [1,3]. Some interventions to identify and reduce missed opportunities for vaccination that have been published include education for patients and parents, electronic notices in the electronic medical records for providers, provider performance reports, patient outreach through postcards, phone calls and home visits, and manual screening of all patients’ vaccination records [4].

In a pharmacy, before a vaccine is administered, a vaccine screening form is routinely completed by all patients to identify contraindications to vaccination [5]. Screening forms contain demographic and health questions and are traditionally used by healthcare providers to determine if a patient is contraindicated to receive the requested vaccine. However, it is possible that a contraindication to one vaccine may be an indication for another. For example, a patient who checked they were immunocompromised would be contraindicated from receiving a live vaccine, but would likely be indicated for the pneumococcal vaccines. In addition, even when the requested vaccine is not contraindicated, the form may indicate there are still other vaccines that may be recommended for the patient. A standardized vaccine screening checklist from the Immunization Action Coalition (IAC) and endorsed by the CDC can be found here: http://www.immunize.org/catg.d/p4065.pdf (Table 1) [6]. There are previous data on the successful use of proprietary forms developed by individual pharmacies to screen and assess patients for vaccination needs; however, to our knowledge, there are no published reports evaluating the use of standard CDC endorsed checklist questions on a pharmacy-based vaccine screening form to not only identify contraindications to vaccination, but also possible vaccination opportunities [7,8]. The CDC has developed additional tools, separate from the screening questionnaire, to identify other indications, such as travel, health conditions, occupation, and lifestyle for vaccinations [9,10].

We believe that vaccine screening forms can be used to identify potential opportunities to vaccinate and make proactive vaccine recommendations based on a uniform set of questions to be answered for every patient being vaccinated at the pharmacy. By retrospectively assessing the forms currently being used in a variety of community pharmacy practice settings, we hope to (1) create a standard set of recommendations based on questions on the screening form that are likely to yield a vaccine indication; and (2) categorize potential opportunities for vaccination, within the population that receive vaccines from the pharmacy, using the screening form questions.

## 2. Materials and Methods

### 2.1. Data Source and Study Sample

This was a retrospective cross-sectional study evaluating one year of data from 11 community pharmacies (6 in California [CA] and 5 in Michigan [MI]). These pharmacies were a convenience sample of pharmacies that also participated as demonstration pharmacies for a more comprehensive American Pharmacists Association/CDC project (Cooperative Agreement Number, IH23IP000984, funded by the CDC) related to immunization registry reporting occurring concurrently with this study. The participating pharmacies represent different areas of community pharmacy practice, including four chain (stand alone, mass merchandiser, and grocery store) and seven independent pharmacies.

All completed vaccine screening forms from 1 June 2015 through 31 May 2016 were gathered from each pharmacy and de-identified data were collected and analyzed using patients’ responses to screening questions (Table 1) and demographic questions asked on the form. Data collected included basic demographics, dichotomous answers to screening questions, and vaccine(s) administered during that visit. Screening forms also serve as vaccine administration records, noting which vaccines were administered during that visit. A comorbidity is considered an answer of “yes” to questions #4, #5, or #6 in Table 1. Data were analyzed using descriptive statistics, and a comparison between the two states was made using Fisher’s test with adjusted *p*-values using the Bonferroni correction. This study was approved by the Institutional Review Board of Chapman University.

### 2.2. Determining Vaccine Indications from Screening Forms

The Advisory Committee on Immunization Practices (ACIP) immunization schedules available as of May 2016 were used to forecast a vaccine needed using the demographic and screening question responses available [11,12]. Only vaccine indications that correlated directly with demographic items or questions on the screening forms were included in the final analysis.

## 3. Results

### 3.1. Study Population

There were a total of 8669 vaccine screening forms (or patient encounters) that represented 9804 administered vaccines, with 10.6% (*n* = 917) of patients receiving more than one vaccine at their visit to the pharmacy (Table 2). The total number of vaccines administered by a single pharmacy over a 1-year period varied from 101 to 2162 vaccines. The majority of the vaccinations that were administered by the pharmacies were influenza vaccines (74.5%; *n* = 7307). The average age of the pharmacy vaccine recipients was 53 years old (range <1–108 years old; median = 56), with 59.4% identifying as female.

### 3.2. Frequency of CDC Recommended Questions Asked

Evaluation of the current use of screening forms by the 11 pharmacies in this project revealed that 7 of the 11 pharmacies asked all 10 of the CDC recommended vaccine screening questions on their screening forms. All the chain (*n* = 2), mass merchandiser (*n* = 1), and grocery store (*n* = 1) pharmacies in this study used all of the recommended CDC screening questions. The independent pharmacies that did not use all the questions, used between 2 to 8 of the questions recommended by the IAC and CDC. The one question that was used by all 11 pharmacies was related to inquiring about allergies (question #2 on Table 1).

### 3.3. Potential Recommended Vaccinations Based on Vaccine Screening Form Responses

We identified that patients who receive vaccination from the pharmacy have at least one additional vaccine, besides the influenza vaccine, that may be recommended for them based on their answers, or demographic information, from the vaccine contraindication screening form. Using the age (or date of birth) from the demographics section of the vaccine contraindication screening forms, one can identify an indication for zoster, pneumococcal, meningococcal ACWY (MCV4), serogroup B meningococcal (MenB), tetanus, diphtheria & acellular pertussis (Tdap), and human papillomavirus (HPV9) vaccination (Table 3). We were able to identify that 10% (*n* = 759) and 34.6% (*n* = 2615) of patients receiving vaccination(s) at the pharmacy may be indicated for the zoster, or both the zoster and pneumococcal vaccines, respectively. Of the 2615 patients that may be indicated for the zoster and pneumococcal vaccines, 952 (36.4%) patients received one or both of those vaccines during their pharmacy vaccine encounter(s) within this study period (Table 3 and Table 4).

The screening forms that inquired about chronic conditions were able to identify the potential need for pneumococcal vaccine in 13.9% (*n* = 977) of the study population. Within this number of patients with chronic conditions that are risk factors for pneumococcal disease, 210 out of the 977 (21.5%) received a pneumococcal vaccine during their pharmacy vaccine encounter(s) within this study period (Table 3 and Table 4).

A group of young males aged 11–21 years old (2.6%, *n* = 195) and females aged 11–26 years old (7.8%, *n* = 592) were identified as patients who should be assessed for HPV9, MCV4, MenB, and Tdap vaccination. We found that only 16.4% (*n* = 32) of the males and 15.9% (*n* = 94) of the females indicated for vaccines received at least one of the recommended vaccines during their pharmacy vaccine encounter within this study period (Table 3 and Table 4). Pregnancy with no determination of trimester was assessed on 10 of 11 pharmacy screening forms, which represented only 0.47% (*n* = 41) of the study population. Of the 41 pregnant women, 9 (22%) received a Tdap vaccine during their pharmacy vaccine encounter(s) within this study period.

Comparing CA and MI for possible recommended vaccinations based on vaccine screening form questions, the only indication that was significantly different (*p* = 0.04199) between the populations was for those adults ≥65 years of age in CA (Table 3). When testing for differences between the two states for those who actually received any of the possibly recommended vaccines during their vaccine encounter at the pharmacy, CA patients ≥65 years, females 11–26 years of age, and those with comorbidities were significantly more likely to receive at least one of the potential vaccines indicated for them (Table 4).

## 4. Discussion

Our study suggests that the vaccine indications that can be inferred from the vaccine contraindication screening form answers were based mostly on the patient’s age, obtained from the demographics section of the pharmacy screening form. For adults, these age-based vaccine indications are for the two pneumococcal vaccines and the zoster vaccines. According to key findings from the CDC’s 2015 Surveillance of Vaccination Coverage among Adult Populations in the United States (U.S.), although there was modest gain in the coverage for pneumococcal and zoster vaccinations compared to the previous year, the coverage is still low and many adults remain unvaccinated with recommended vaccines [13]. In children and adolescents, the age-based vaccine indications that can be inferred from the screening form responses are for the HPV9, MCV4, MenB, and Tdap vaccines. In 2016, the CDC estimated that only 43.4% of teens had received the complete recommended HPV vaccination series and that among teens aged 13–17 years, coverage with two or more MenACWY doses was 39.1% [14]. The CDC also recommends that clinicians consistently recommend and simultaneously administer Tdap, MenACWY, and HPV vaccines at age 11–12 years as a method to increase coverage of these vaccines. In both California and Michigan, state law allows pharmacists to administer all adult and adolescent vaccines without a prescription from a physician [15]. For example, when teens present for influenza vaccination, reviewing the patient’s response to the vaccine contraindication screening form allows identification of other vaccination needs to discuss with the patient and parent.

When looking at actual questions on the screening forms, we were able to identify that 4 out of the 10 questions are likely to yield a vaccine recommendation. An answer of “yes” to three of these questions (#4, #5, or #6) elicits the possible need for one, or both, of the pneumococcal vaccines (Table 1). These questions ask about the presence of comorbidities such as chronic illnesses (chronic heart, liver, kidney, lung, diabetes) and impaired immune function due to disease or medications. Although the intention of these three questions is to avoid administering a live vaccine to patients with these risk factors, the content of these three questions essentially also asks the patient to confirm if they have factors that increase their risk for getting pneumococcal disease, and therefore should be recommended pneumococcal vaccination. We were able to identify that 13.9% of our study sample with comorbidities (i.e., answering “yes” to questions #4, #5, or #6) would be indicated for the pneumococcal vaccine. However, in this high-risk group, national data suggest that only approximately 25% of adults less than 65 years of age with an indication for pneumococcal vaccination report ever having been vaccinated with pneumococcal vaccine [13]. Using a patient’s response to the screening form questions that ask about comorbidities can identify these patients that often go unvaccinated. Even the CDC states that their screening questions are for precautions and contraindications to vaccines, not indications [6]. In the pharmacies in our sample, only the screening form was used and not the CDC’s questionnaire to determine the recommended vaccines. Thus a large opportunity to identify patients potentially in need of recommended vaccines is lost.

The number of patients with comorbid conditions indicative of the need for immunization in our study is likely an underestimation due to several possible screening form discrepancies: (1) At one pharmacy, patients may have left the question blank because it asked it as a free-response question instead of a yes/no; (2) at another pharmacy in our study sample, they did not ask about comorbidities at all; and (3) some pharmacies had procedures that instructed patients not to complete the questions that ask about comorbidities because they were there for an inactivated vaccine and having a comorbidity would generally not be a contraindication to receiving inactivated vaccines. To increase the detection of comorbidities, pharmacies can also use inferred diagnoses (i.e., using the medications a patient takes to infer their diagnoses) from their pharmacy dispensing records. Obtaining an inferred diagnosis for comorbidities was not possible in our sample as a current medication list was not included in the pharmacy screening questionnaires.

The small number of pregnant women identified in our pharmacy population may be expected, as the literature suggests that the majority of pregnant women tend to receive their Tdap (96%) and influenza (97.3%) vaccine in a traditional healthcare setting, such as their OB/GYN or family doctor [16,17]. However, pharmacists should still incorporate a vaccine needs assessment for pregnant women, as the U.S. maternal vaccination rate with Tdap and influenza vaccines is suboptimal. During the 2017–2018 influenza season, only 49.1% of pregnant women received influenza vaccination, 54.4% with a live birth received Tdap during pregnancy, and 32.8% received both recommended vaccines [18]. Other vaccines may be indicated for patients of different ages, medical conditions, occupations, and lifestyles, but it would require the pharmacist to elicit more information from the patient.

Adult vaccination rates are still below the Healthy People 2020 target goals, and pharmacy-based immunization screening forms can identify opportunities to improve immunization rates. The zoster vaccine goal is set very low at 30% compared to the other vaccines (e.g., 90% for pneumococcal), and its coverage is currently only at 33.4% using the now non-preferred live zoster vaccine [19]. Using pharmacy screening forms to infer indications for vaccinations is another method that can identify patients who should be vaccinated to increase coverage from vaccine-preventable diseases and help reach our Healthy People 2020 goals. The CDC and other organizations have developed additional tools for screening, which may increase the chances of identifying missed vaccine opportunities, but may also increase paperwork and workflow burden for pharmacists and patients [9,10,20]. The additional CDC questionnaire asks about other risk factors, such as if they are a healthcare worker, a man who has sex with men, a college student, homeless, are planning travel, etc. Since the majority of the vaccinations that were administered by the pharmacies were influenza vaccines (74.5%), and influenza is recommended for everyone 6 months and older, this increases the pharmacists’ potential to identify and make recommendations on other vaccine needs on, at least, an annual basis.

Across the board for all vaccines that were identified as potentially indicated, only 20–37% of those indicated for the vaccine received that recommended vaccine during the pharmacy encounter (Table 4). These results indicate that possibly 63–80% of the potential vaccine opportunities that are identified using the screening form are true opportunities for the pharmacist to initiate a conversation with the patient to assess for other needed vaccines.

While the age and pregnancy status of patients in CA and MI were similar, we did note a statistically significantly greater percentage of the sample in CA being 65 years or older compared to MI. This project, however, was not designed to detect and determine the reason for differences in the populations vaccinated between the two states.

Published studies have centered on the success pharmacists have had in targeted vaccine interventions to improve immunization rates, but all patients who come in for any vaccinations should be screened for all additional vaccine needs [21,22,23,24,25]. Knowing the potential opportunities for vaccination allows for the pharmacist or provider to directly assess the status of that specific vaccine with the patient through open conversation or review of their records. A limitation of this study is that the patients included in this study were from a convenience sample of eleven pharmacies limited to Southern California and Michigan who agreed to participate. However, both chain and independent pharmacies were included. In addition, the screening forms used by the chain pharmacies in this project represent at least 13,378 pharmacies that use these forms across the United States, amplifying the impact of this opportunity.

## 5. Conclusions

Our data indicate that pharmacists can identify potential immunization opportunities proactively by using their current vaccine contraindication screening form. Age and comorbid conditions are routinely collected by pharmacy-based immunization programs, but not all are using them the same way, and none in this sample were actively using them to discover indications for vaccination. Additionally, several occupational and lifestyle questions are missing from the pharmacy screening form that are included in the CDC questionnaire. Therefore, all pharmacies should use the standard CDC screening questions, as well as the questionnaire, and ensure all questions are filled out completely by the patient so that any vaccination recommendations can be communicated to the patient at the same time. Pharmacies should also use their immunization information system and pharmacy dispensing system to proactively identify patients for vaccination. This leads to the identification of missed vaccination opportunities, which may decrease the need for multiple visits, assist with follow-up, and increase vaccine rates.

## Figures and Tables

**Table 1:** Immunization Action Coalition (IAC) and Centers for Disease Control and Prevention (CDC) recommended questions when screening for vaccine contraindications and precautions.

Question Number	Question
1	Are you sick today?
2	Do you have allergies to medications, food, a vaccine component, or latex?
3	Have you ever had a serious reaction after receiving a vaccination?
4	Do you have a long-term health problem with heart disease, lung disease, asthma, kidney disease, metabolic disease (e.g., diabetes), anemia, or other blood disorders?
5	Do you have cancer, leukemia, HIV/AIDS, or any other immune system problem?
6	In the past 3 months, have you taken medications that affect your immune system, such as prednisone, other steroids, or anticancer drugs; drugs for the treatment of rheumatoid arthritis, Crohn’s disease, or psoriasis; or have you had radiation treatment?
7	Have you had a seizure or a brain or other nervous system problem?
8	During the past year, have you received a transfusion of blood or blood products, or been given immune (gamma) globulin or an antiviral drug?
9	For women: Are you pregnant or is there a chance you could be pregnant during the next month?
10	Have you received any vaccinations in the past 4 weeks?

These are the questions recommended by the IAC and the CDC to screen patients for precautions and contraindications to vaccines, adopted into a table format [5].

**Table 2:** Patient encounter demographics.

	*N* (%)	CA	MI
Patient encounters/screening forms	8669	3163 (36.5)	5506 (63.5)
Total number of vaccines administered	9804	3937 (41.8)	5867 (59.8)
Patient encounters receiving >1 vaccine	917 (10.6)	581 (63.4)	336 (36.6)
Average age (years) ^	52.8 ± 20.8 Median = 56	53.5	52.4
Patients with self-reported co-morbidities *	977 (13.9)	411 (42.1)	566 (57.9)

^ 7555 forms provided dates of birth or valid dates of birth. * Only 7018 forms asked about co-morbidities, indicating % of those asked. CA, California; MI, Michigan.

**Table 3:** Possible recommended vaccinations based on vaccine screening form.

Indication	*N* (%)	*N* (%) CA ^+^	*N* (%) MI ^#^	Fisher’s Test Bonferroni Adjusted *p-*Value	Vaccine Recommendation	Screening Form Item
**AGE**						
60–64 years ^	759 (10.0)	319 (10.3)	440 (9.9)	*p* = 1.0	ZVL	Demographics
≥65 years ^	2615 (34.6)	1160 (37.4)	1455 (32.7)	*p* = 0.04199	PCV13, PPSV23, ZVL	Demographics
11–21 (males) ^	195 (2.6)	98 (3.2)	97 (2.2)	*p* = 0.14508	HPV, MCV4, MenB, Tdap	Demographics
11–26 (females) ^	592 (7.8)	235 (7.6)	357 (8.0)	*p* = 1.0	HPV, MCV4, MenB, Tdap	Demographics
**OTHER CONDITIONS**						
Patient with comorbidities *	977 (13.9)	411 (13.2)	566 (12.7)	*p* = 1.0	PCV13, PPSV23	Questions: #4, #5, #6
Pregnant women	41 (0.47)	11 (0.4)	30 (0.7)	*p* = 1.0	Tdap	Question: #9

^ 7555 forms provided dates of birth or valid dates of birth. * Only 7018 forms asked about co-morbidities, indicating % of those asked. ^+^ 3103 forms in CA that provided dates of birth or valid dates of birth. ^#^ 4452 forms in MI that provided dates of birth or valid dates of birth. Key: ZVL = zoster vaccine live; PCV13 = pneumococcal conjugate; PPSV23 = pneumococcal polysaccharide; HPV = human papillomavirus; MenB = serogroup B meningococcal; Tdap = tetanus, diphtheria & acellular pertussis; MCV4 = meningococcalACWY.

**Table 4:** Patients that received any of the indicated vaccine(s) during their encounter.

Indication	Vaccine	*N* (%)	Total Patients Receiving Either Vaccine(s) during Encounter *N* (%)	*N* (%) CA	*N* (%) MI	Fisher’s Test Bonferroni Adjusted *p*-Value
**AGE**						
60–64 years ^	ZVL	759 (10.0)	148 (19.5)	72 (48.6)	76 (51.4)	*p* = 1.0
≥65 years ^	PCV13, PPSV23, ZVL	2615 (34.6)	952 (36.4)	533 (56.0)	419 (44.0)	*p* < 0.001
11–21 (males) ^	HPV, MCV4, MenB, Tdap	195 (2.6)	32 (16.4)	20 (62.5)	12 (37.5)	*p* = 1.0
11–26 (females) ^	HPV, MCV4, MenB, Tdap	592 (7.8)	94 (15.9)	77 (81.9)	17 (18.1)	*p* = 0.00534
**OTHER CONDITIONS**						
Patient with comorbidities *	PCV13, PPSV23	977 (13.9)	210 (21.5)	113 (53.8)	97 (46.8)	*p* = 0.02587
Pregnant women	Tdap	41 (0.47)	9 (22)	7 (77.8)	2 (22.2)	*p* = 0.08254

^ 7555 forms provided dates of birth or valid dates of birth. * Only 7018 forms asked about co-morbidities, indicating % of those asked. Key: ZVL = zoster vaccine live; PCV13 = pneumococcal conjugate; PPSV23 = pneumococcal polysaccharide; HPV = human papillomavirus; MenB = serogroup B meningococcal; Tdap = tetanus, diphtheria & acellular pertussis; MCV4 = meningococcalACWY.
